# The Effects of Online Access to General Practice Medical Records Perceived by Patients: Longitudinal Survey Study

**DOI:** 10.2196/47659

**Published:** 2023-06-02

**Authors:** Rosa R L C Thielmann, Ciska Hoving, Jochen W L Cals, Rik Crutzen

**Affiliations:** 1 School for Public Health and Primary Care Faculty of Health, Medicine and Life Sciences Maastricht University Maastricht Netherlands

**Keywords:** electronic health records, personal health records, medical records, patient access to records, patient portals, patient participation, informed decision-making, patient empowerment

## Abstract

**Background:**

Patient online access to medical records is assumed to facilitate patient empowerment and advance patient-centered health care. However, to date, the actual effects of online access to medical records perceived by patients and other outcomes are insufficiently empirically tested.

**Objective:**

This study aimed to investigate the effects of online access to medical records on patient empowerment, informed decision-making, and the patient-provider relationship perceived by patients.

**Methods:**

A nationwide, 2-wave, longitudinal survey study was conducted among Dutch adults (N=2402). Linear regression analyses were performed. In model 1, the perceived effects of online access to medical records (measured at T1 [first measurement; July 2021]) on 16 outcomes (measured at T2 [second measurement; January 2022]), which were associated with the use of online access to general practice medical records in previous research, were investigated. Model 2 included sociodemographic factors and patient characteristics as confounders.

**Results:**

Users indicated more strongly than nonusers that online access to medical records would increase their participation in health care, improve the relationship with their general practitioner, and support informed decision-making. These results were robust when adjusted for the influence of confounders. Effect sizes were very small, with unstandardized regression coefficients (B) ranging between −0.39 and 0.28. Higher digital and health literacy were associated with higher ratings of almost all effects.

**Conclusions:**

Online access to medical records has the potential to empower patients and foster informed decision-making among patients. The effects in this study were small but might grow over time. Other factors, such as the attitude of general practitioners toward online access to medical records, might moderate these effects. The results indicate that the potential benefits of online access to medical records might be unevenly distributed. We suggest future exploration of the conditions under which online access to medical records can improve health care system functioning and efficiency without increasing health inequality.

## Introduction

In an increasing number of countries worldwide, patients are being offered online access to their medical records. The idea behind this is to improve health care system functioning and efficiency by fostering patient empowerment and to advance patient-centered health care [1]. Conceptualizations of these terms often illustrate an ideological shift in the patient-provider relationship from paternalistic to increasingly patient participation–based health care in which communication and respecting the patient’s voice are key values [2].

Online access to medical records has already become an integral part of many health care systems. In the United States, for example, the “OpenNotes” initiative began over a decade ago [3] and now facilitates access to the medical records of roughly 41 million patients [4]. Moreover, in Nordic countries, most patients are already offered online access to their medical records [5]. In the Netherlands, patients became legally entitled to access parts of their general practice medical records electronically in July 2020. Access is mainly facilitated via online patient portals that are directly tethered to the electronic medical records held by the general practice. Patients can currently view medication and allergy lists, medical notes, and diagnostic test results [6].

Positive effects from online access to medical records are thought to arise partly due to increased personal health care knowledge (eg, of a health condition or treatment) for patients [7]. This may facilitate informed health care decision-making [8]. Informed decision-making is the process in which a patient comes to a decision that is based on relevant and good quality knowledge, which reflects the patient’s values, and that can be subsequently implemented [9,10]. Such a decision can prevent the experience of “decisional conflict” for patients, which is the experience of uncertainty or regret about their decision [11,12], and foster treatment adherence [13].

The results of a previous interview study exploring Dutch patients’ expectations regarding online access to their medical records pointed to the possible effects [14]. Participants imagined that online access to their medical records would give them a better overview about their health care and appointments, and that it would promote trust in and improve communication with their general practitioner (GP) [14]. They expected increased self-efficacy for actions like accessing test results independently and imagined this to result in fewer telephone calls with the general practice office. Participants indeed anticipated an empowered role in the GP-patient relationship and in health care decision-making [14]. However, they also imagined distress and anxiety when reading sensitive, incomprehensible, or incorrect information in their medical records [14]. Patients raised similar concerns in other studies [15,16]. Connected to this are GPs’ worries that patient online access to medical records could increase their workload, as they might have to answer additional questions and resolve misunderstandings [17].

Naturally, monitoring effects is necessary to evaluate the potential impact of online access to medical records as a public health measure. Moreover, exploration of effects is important to accurately inform patients. Patients’ interest in using online health information is strongly predicted by their expectations of benefits from it [18]. Moreover, beliefs about effects were found to be highly relevant for patients to make an informed decision about whether they want to use online access to their medical records (regardless of the outcome of that decision) [19]. Despite efforts to understand the complex process of how online access to medical records might impact patients and health systems, systematic reviews described the evidence to draw conclusions on the actual influence on patient empowerment and decision-making as insufficient [17,20,21]. Therefore, this study investigated the effects of patient online access to medical records on patient empowerment, informed decision-making, and the GP-patient relationship perceived by patients.

## Methods

### Research Design

This study was part of a larger project with a longitudinal cohort design. The project was preregistered in the Open Science Framework (OSF; 3gnx2) [22]. Data were collected via an online survey. For this study, data about participants’ use of online access to their medical records from the first measurement (July 2021 [T1]) and perceived effects of online access to their medical records from the second measurement (January 2022 [T2]) were analyzed. Data collected in this study were pseudonymized before analysis, meaning that the researchers could not identify specific persons from the data set [23]. We followed the STROBE (Strengthening the Reporting of Observational Studies in Epidemiology) guidelines for observational research to prepare this article [24]. The survey, analysis scripts, output of the analysis including exact *P* values, and nonidentifiable data are available or can be requested at OSF [22].

### Ethics Approval

The project was approved by the Maastricht University Faculty Research Ethics Committee (approval number: FHML-REC/2021/071).

### Participants and Procedure

Participants were recruited by the Dutch ISO-certified internet research agency *Flycatcher* [25] from among its panel members. We calculated the desired sample size for T1 based on the desired number of participants remaining at the end of the larger project and the expected dropout rate of 30%-35% between measurements. As we could not infer the effect size from earlier research, we assumed a small effect size of Cohen *d*=0.2, a margin of error (half-width) of 0.1, and a confidence level of 95%. These assumptions were included in the sample size calculation using the “ufs” package [26] in R [27]. We aimed for a total sample size of 3460 participants for T1 and expected 2336 participants to remain at T2.

Adult patients residing in the Netherlands with at least one contact with a general practice within the past 6 months were eligible for inclusion, as we were interested in recent experiences. The research agency identified these panel members and subsequently invited a sample representative for this group based on age, gender, education, and region within the Netherlands to participate in this study. At T1, within a 1-week time span, the sample received 1 invitation and 2 reminders via email. All participants who completed the survey at T1 were invited again to participate in the T2 measurement via 1 invitation. In accordance with the principle of data minimization [23], no reminders were sent at T2 as the response following initial invitation was already sufficient to reach the required sample size and we did not want to unnecessarily burden participants. Informed consent was obtained online. Completing each survey took 15 minutes on average. Participants were reimbursed in the form of panel points worth about 2 euros (2.14 US dollars), which could be exchanged for gift vouchers.

Survey items relevant to this study concerned (1) sociodemographic characteristics, (2) the predictor variable (ie, use of online access to medical records at T1), and (3) dependent variables (ie, beliefs about the effects of online access to medical records at T2). Pretesting of the survey took place with both native and second-language Dutch speakers.

### Measurements

#### Sociodemographic Characteristics

The following sociodemographic characteristics were assessed at T1 as earlier research indicated a potential relationship with the use of online access to medical records: age [28], gender identity [29], educational level, migration background, region [30], digital and health literacy [31], presence of a chronic illness, health status [32], and whether the GP was ever visited due to a psychological complaint [33]. Sociodemographic variables were used to both describe the characteristics of the study population and investigate the potential confounding impact of these variables in the main analysis owing to possible relationships indicated by earlier research. The highest completed educational level was categorized as low (eg, primary education), intermediate (eg, secondary vocational education), and high (eg, university education) [34]. A participant was considered a migrant if the participant was born abroad [35]. Digital literacy, defined by the American Library Association as “the ability to use information and communication technologies to find, evaluate, create, and communicate information, requiring both cognitive and technical skills” [36], was assessed with 5 items from the Dutch “Quick scan digital skills” measurement tool. It was developed by the Dutch Centre of Expertise on Health Disparities to identify patient’s digital literacy in general practice [37]. Items asked, for example, “Do you sometimes use an app?” and answer options (scores) were “no” (0), “with help of, for example, family or friends” (2), and “yes” (4). All item scores were summed, divided by 5, and multiplied by 25. Sum scores range from 0 to 100, with a higher score indicating higher digital literacy. The World Health Organization describes health literacy as the skills individuals need to gain access to, understand, and use information in ways that promote their health [38]. To assess health literacy, 6 items were chosen from the HSL-EU-Q47 [39] that cover all cognitive domains deemed necessary to handle health information within the health care setting [40]. This choice was made to obtain a multifaceted yet concise indication of health literacy. Items were formulated as questions (eg, “How easy would you say it is to find information on treatment of illnesses that concern you?”). Health literacy sum scores were computed by summing the responses “very easy” and “easy” coded as 1, and “difficult” and “very difficult” coded as 0 [41]. Scores range from 0 to 6, with a higher score indicating higher health literacy.

#### Predictor Variable: Use of Online Access to Medical Records

Use was defined as having accessed medical records from general practice online at least once at T1. After participants received written and video explanations about what online access constitutes, the survey asked, “Have you ever accessed your GP medical records online?” with answer options “no” and “yes.”

#### Dependent Variables: Beliefs About the Effects of Online Access to Medical Records

At T2, a set of 16 different items was used to assess beliefs about the effects of online access to medical records, resulting in 16 distinct outcome variables. Content of the items was derived from expectations mentioned by patients in a preceding interview study [14]. Those expectations were operationalized by following instructions on measuring instrumental attitude belief expectations from the PsyCoRe repository [42]: items all began with “By using online access, …” followed by the possible effect. Bidimensional 7-point Likert scales were embedded in the statements, with the left anchor being the lesser/lower/worse assessment and the right anchor being the more/higher/better assessment of a belief (eg, “… I have way less (1) – way more (7) overview about my health care”). Eight items assessed expected practical changes in health care, 5 items assessed the expected impact on affective outcomes and the GP-patient relationship, and 3 items assessed the expected influence on informed health care decision-making. The questions used to assess the outcome variables can be found in Multimedia Appendix 1.

### Analysis

Analyses were performed in SPSS 28 (IBM Corp). Descriptive statistics were used to characterize the study population. Chi-square tests were performed to determine whether the proportion of participant characteristics measured with categorical variables was equal, and *t* tests were performed to determine whether means of characteristics measured with continuous variables were equal between participants who had ever or had never used online access to their medical records. Correlations were explored and categorized as small (0.10 ≤ *r* < 0.30), medium (0.30 ≤ *r* < 0.50), and large (*r* ≥ 0.50) [43]. To investigate the perceived effects of online access to medical records for patients, a (multiple) linear regression analysis was conducted for each effect separately. To evaluate how well the use of online access to medical records could explain an anticipated effect, hierarchical regression was performed with 2 blocks. Model 1 contained only the predictor variable (use of online access to medical records). Model 2 additionally contained potential confounders, that is, sociodemographic variables (categorical variables with k levels were transformed into k−1 variables each with 2 levels). Acknowledging that results following stepwise entry techniques are influenced by random variation in the data and therefore provide a false sense of accuracy (ie, they rarely provide replicable results if the model is retested) [44], we used forced entry methods in both models. Unstandardized regression coefficients were reported to interpret the impact of online access to medical records and each confounder on beliefs about effects. To control for multiple testing, we used the Benjamini and Hochberg linear step-up method [45]. Using Excel (Microsoft Corp), we calculated adjusted significance levels for each effect.

## Results

### Patient Characteristics

The dropout rate between the study waves was 29.4% (1002/3404), and the characteristics of individuals who dropped out did not differ from those who remained. In total, 2402 participants completed the survey at T2 and were included in the analyses. At T1, 803 (33.4%) participants had made use of online access to their medical records at least once. The mean age of the participants was 52.59 years, and 48.0% (1152/2402) were female. The educational level was categorized as intermediate for 47.0% (1129/2402) and high for 25.5% (613/2402) of the participants. Patient characteristics did not differ between ever users and never users, besides digital literacy (*t*_2400_=−4.125; *P*<.001), chronic disease presence (*χ*^2^_3,2402_=19.42; *P*<.001), and visiting the GP due to a mental health complaint (*χ*^2^_2,2402_=10.43; *P*=.02). Table 1 shows the characteristics of the study population.

**Table 1:** Participant characteristics assessed at T1 (first measurement; July 2021).

Variable		Total (N=2402)	Ever users (n=803)	Never users (n=1599)	*P* value^a^
Age (years), mean (SD)		52.59 (16.39)	52.87 (16.09)	52.46 (16.55)	.56
**Gender, n (%)**					.88
	Female	1152 (48.0)	388 (48.3)	764 (47.8)	
	Male	1244 (51.8)	413 (51.4)	831 (52.0)	
	Another gender/nonbinary	6 (0.2)	2 (0.2)	4 (0.3)	
**Education level, n (%)**					.12
	Low	660 (27.5)	202 (25.2)	458 (28.6)	
	Intermediate	1129 (47.0)	399 (49.7)	730 (45.7)	
	High	613 (25.5)	202 (25.2)	411 (25.7)	
Migration background, n (%)		106 (4.4)	35 (4.4)	71 (4.4)	.93
Health literacy (range 0-6), mean (SD)		5.47 (1.17)	5.47 (1.26)	5.47 (1.12)	.43
Digital literacy (range 0-100), mean (SD)		92.57 (16.81)	94.56 (14.75)	91.57 (17.67)	<.001
Chronic disease presence, n (%)		977 (40.7)	374 (46.6)	603 (37.7)	<.001
GP^b^ visit due to a psychological complaint (ever), n (%)		881 (36.7)	330 (41.1)	551 (34.5)	.02

^a^Testing of means was performed with *t* tests, and testing of frequency distribution was performed with Pearson chi-square tests.

^b^GP: general practitioner.

### Linear Regression of the Use of Online Access to Medical Records and Patient Characteristics for Beliefs About the Impact of Online Access to Medical Records

#### Perceived Effects of Online Access to Medical Records

Results of linear regression analyses of determinants for beliefs about the effects of online access to medical records are shown in Tables 2-5.

Users were more likely to perceive online access to (1) cause practical changes in GP health care, (2) have affective benefits and improve the relationship with their GP, and (3) support informed decision-making. These effects were robust even when sociodemographic factors and patient characteristics were included in model 2. All effect sizes were rather small, with unstandardized regression coefficients (B) ranging between −0.39 and 0.28 (eg, on a 7-point scale, users rated the potential impact of online access on being better able to prepare consultations with the GP 0.1 points higher than nonusers).

First, compared with nonusers, users perceived online access to impact 5 of the 8 effects measured in the domain of *practical changes in their GP health care*. They indicated more strongly that online access would lead to (1) more personal contact with the GP and the practice staff, (2) more consultations, (3) more telephone calls with the GP or the practice assistant, (4) more time investment in health care, and (5) an increased ability to prepare consultations with the GP. There were no differences between users’ and nonusers’ perceptions of the impact of online access to their medical records on their overview of health care and appointments, and their ability to correct mistakes in the medical record.

Second, across all 5 measured items, users reported that online access would lead to *improvements* *in affective outcomes and the GP-patient relationship*. Specifically, compared with nonusers, users indicated more strongly that online access would lead to (1) feeling less overwhelmed, (2) feeling less anxious, (3) better communication with the GP, (4) more patient involvement, and (5) more equal-feeling conversations with the GP.

Third, across all 3 items, users indicated more than nonusers that online access would support *informed decision-making*. Users more strongly expressed that online access leads to (1) having more information to make decisions about their health, (2) an increased ability to make decisions about health that align with own values, and (3) an increased ability to make decisions about health in general.

**Table 2:** Multiple linear regression analyses of determinants for the effects of online access to medical records (overview of health care, overview of appointments, correct mistakes, and feeling overwhelmed; N=2402).

Variable			Overview of health care		Overview of appointments		Correct mistakes		Feeling overwhelmed
Score^a^, mean (SD)			5.53 (1.19)		5.45 (1.27)		5.48 (1.27)		3.44 (1.53)
**Model 1**									
	Online access, B^b^ (95% CI)	0.10 (0.00 to 0.20)		0.08 (−0.03 to 0.18)		0.05 (−0.06 to 0.16)		−0.21^c^ (−0.34 to −0.08)	
	*R*^2^ (adjusted *R*^2^)	0.00 (0.00)		0.00 (0.00)		0.00 (0.00)		0.00 (0.00)^c^	
**Model 2**									
	Online access, B (95% CI)	0.06 (−0.04 to 0.16)		0.05 (−0.06 to 0.15)		0.00 (−0.11 to 0.11)		−0.16^c^ (−0.29 to −0.04)	
	Female vs other, B (95% CI)	−0.19 (−1.13 to 1.37)		−0.41 (−1.42 to 0.60)		0.05 (−0.95 to 1.05)		0.08 (−1.13 to 1.28)	
	Female vs male, B (95% CI)	−0.10 (−0.19 to 0.00)		−0.06 (−0.17 to 0.04)		−0.17^c^ (−0.27 to −0.07)		0.03 (−0.10 to 0.15)	
	Low ES^d^ vs medium ES, B (95% CI)	0.13^c^ (0.02 to 0.25)		0.14^c^ (0.01 to 0.26)		0.13^c^ (0.01 to 0.26)		−0.36^c^ (−0.51 to −0.21)	
	Low ES vs high ES, B (95% CI)	0.12 (−0.02 to 0.25)		0.10 (−0.05 to 0.25)		0.09 (−0.05 to 0.24)		−0.59^c^ (−0.76 to −0.41)	
	No vs minimum of one visit to the GP^e^ due to a mental health complaint, B (95% CI)	0.04 (−0.06 to 0.13)		0.01 (−0.10 to 0.12)		0.12^c^ (0.02 to 0.23)		0.01 (−0.12 to 0.13)	
	No vs minimum of one chronic disease, B (95% CI)	−0.04 (−0.15 to 0.06)		−0.06 (−0.17 to 0.04)		0.03 (−0.07 to 0.14)		−0.11 (−0.24 to 0.02)	
	Born in NL^f^ vs migrant, B (95% CI)	−0.01 (−0.24 to 0.24)		−0.05 (−0.30 to 0.20)		−0.11 (−0.36 to 0.13)		0.15 (−0.14 to 0.45)	
	Age, B (95% CI)	0.00 (0.00 to 0.00)		0.00 (0.00 to 0.01)		0.00^c^ (0.00 to 0.01)		0.00 (−0.01 to 0.00)	
	Digital literacy, B (95% CI)	0.12^c^ (0.09 to 0.15)		0.09^c^ (0.06 to 0.12)		0.11^c^ (0.08 to 0.14)		−0.07^c^ (−0.11 to −0.03)	
	Health literacy, B (95% CI)	0.08^c^ (0.04 to 0.12)		0.07^c^ (0.03 to 0.11)		0.07^c^ (0.03 to 0.11)		−0.14^c^ (−0.19 to −0.09)	
	*R*^2^ (adjusted *R*^2^)	0.05 (0.05)^c^		0.03 (0.02)^c^		0.04 (0.04)^c^		0.05 (0.05)^c^	

^a^All effects were measured on a scale ranging from 1 to 7.

^b^B: unstandardized coefficient.

^c^Significant value (*P*<.05).

^d^ES: education status.

^e^GP: general practitioner.

^f^NL: the Netherlands.

**Table 3:** Multiple linear regression analyses of determinants for the effects of online access to medical records (feeling anxious, personal contact, number of consultations, and telephone contact; N=2402).

Variable			Feeling anxious		Personal contact		Number of consultations		Telephone contact
Score^a^, mean (SD)			3.03 (1.46)		3.89 (1.54)		4.03 (1.40)		3.97 (1.43)
**Model 1**									
	Online access, B^b^ (95% CI)	−0.39^c^ (−0.51 to −0.27)		0.28^c^ (0.15 to 0.41)		0.19^c^ (0.07 to 0.30)		0.18^c^ (0.06 to 0.30)	
	*R*^2^ (adjusted *R*^2^)	0.02 (0.02)^c^		0.01 (0.01)^c^		0.00 (0.00)^c^		0.00 (0.00)^c^	
**Model 2**									
	Online access, B (95% CI)	−0.35^c^ (−0.47 to −0.23)		0.31^c^ (0.18 to 0.44)		0.21^c^ (0.09 to 0.33)		0.20^c^ (0.08 to 0.32)	
	Female vs other, B (95% CI)	−0.21 (−1.35 to 0.93)		0.55 (−0.66 to 1.77)		−0.03 (−1.14 to 1.08)		0.24 (−0.91 to 1.38)	
	Female vs male, B (95% CI)	−0.02 (−0.14 to 0.09)		0.10 (−0.03 to 0.22)		0.09 (−0.02 to 0.21)		0.11 (−0.01 to 0.22)	
	Low ES^d^ vs medium ES, B (95% CI)	−0.19^c^ (−0.33 to −0.05)		−0.25^c^ (−0.40 to −0.10)		−0.20^c^ (−0.33 to −0.06)		−0.12^c^ (−0.26 to 0.02)	
	Low ES vs high ES, B (95% CI)	−0.19^c^ (−0.35 to −0.02)		−0.50^c^ (−0.68 to −0.32)		−0.47^c^ (−0.64 to −0.31)		−0.33^c^ (−0.50 to −0.16)	
	No vs minimum of one visit to the GP^e^ due to a mental health complaint, B (95% CI)	0.11 (−0.01 to 0.23)		−0.17^c^ (−0.30 to −0.04)		−0.12^c^ (−0.24 to −0.01)		−0.16^c^ (−0.28 to −0.04)	
	No vs minimum of one chronic disease, B (95% CI)	−0.07 (−0.19 to 0.05)		−0.13 (−0.26 to 0.01)		−0.09 (−0.21 to 0.03)		0.01 (−0.11 to 0.14)	
	Born in NL^f^ vs migrant, B (95% CI)	0.31^c^ (0.03 to 0.59)		0.54^c^ (0.25 to 0.84)		0.20 (−0.07 to 0.48)		0.18 (−0.10 to 0.46)	
	Age, B (95% CI)	0.00^c^ (−0.01 to 0.00)		0.00 (0.00 to 0.01)		0.00 (0.00 to 0.01)		0.00 (0.00 to 0.00)	
	Digital literacy, B (95% CI)	−0.11^c^ (−0.15 to −0.07)		−0.02 (−0.06 to 0.02)		−0.01 (−0.05 to 0.02)		−0.02 (−0.06 to 0.01)	
	Health literacy, B (95% CI)	−0.13^c^ (−0.18 to −0.08)		0.08^c^ (0.03 to 0.13)		0.04 (0.00 to 0.09)		0.06^c^ (0.01 to 0.11)	
	*R*^2^ (adjusted *R*^2^)	0.06 (0.05)^c^		0.04 (0.03)^c^		0.03 (0.02)^c^		0.02 (0.02)^c^	

^a^All effects were measured on a scale ranging from 1 to 7.

^b^B: unstandardized coefficient.

^c^Significant value (*P*<.05).

^d^ES: education status.

^e^GP: general practitioner.

^f^NL: the Netherlands.

**Table 4:** Multiple linear regression analyses of determinants for the effects of online access to medical records (time investment, involvement in health care, equal conversations, and prepare consultations; N=2402).

Variable			Time investment		Involvement in health care		Equal conversations		Prepare consultations
Score^a^, mean (SD)			4.66 (1.23)		5.28 (1.24)		4.74 (1.17)		5.21 (1.15)
**Model 1**									
	Online access, B^b^ (95% CI)	0.13^c^ (0.02 to 0.23)		0.20^c^ (0.09 to 0.30)		0.18^c^ (0.08 to 0.28)		0.12^c^ (0.02 to 0.22)	
	*R*^2^ (adjusted *R*^2^)	0.00 (0.00)^c^		0.01 (0.01)^c^		0.01 (0.00)^c^		0.00 (0.00)^c^	
**Model 2**									
	Online access, B (95% CI)	0.11^c^ (0.01 to 0.22)		0.16^c^ (0.06 to 0.27)		0.16^c^ (0.06 to 0.26)		0.10^c^ (0.00 to 0.20)	
	Female vs other, B (95% CI)	0.34 (−0.65 to 1.32)		0.36 (−0.62 to 1.34)		0.45 (−0.48 to 1.38)		0.81 (−0.10 to 1.73)	
	Female vs male, B (95% CI)	−0.05 (−0.15 to 0.05)		−0.13^c^ (−0.23 to −0.03)		0.00 (−0.09 to 0.10)		−0.04 (−0.14 to 0.05)	
	Low ES^d^ vs medium ES, B (95% CI)	−0.01 (−0.13 to 0.12)		0.04 (−0.08 to 0.16)		0.00 (−0.12 to 0.11)		0.08 (−0.04 to 0.19)	
	Low ES vs high ES, B (95% CI)	−0.11 (−0.25 to 0.04)		−0.01 (−0.15 to 0.13)		0.08 (−0.05 to 0.22)		0.10 (−0.03 to 0.23)	
	No vs minimum of one visit to the GP^e^ due to a mental health complaint, B (95% CI)	−0.13^c^ (−0.23 to −0.02)		−0.08 (−0.19 to 0.02)		−0.07 (−0.17 to 0.03)		−0.05 (−0.15 to 0.05)	
	No vs minimum of one chronic disease, B (95% CI)	0.05 (−0.05 to 0.16)		0.00 (−0.11 to 0.10)		0.03 (−0.07 to 0.13)		0.01 (−0.08 to 0.11)	
	Born in NL^f^ vs migrant, B (95% CI)	0.20 (−0.04 to 0.44)		0.07 (−0.17 to 0.31)		0.11 (−0.11 to 0.34)		0.11 (−0.11 to 0.34)	
	Age, B (95% CI)	0.00 (0.00 to 0.00)		0.00 (0.00 to 0.01)		0.00^c^ (0.00 to 0.01)		0.00^c^ (0.00 to 0.01)	
	Digital literacy, B (95% CI)	0.05^c^ (0.02 to 0.08)		0.12^c^ (0.08 to 0.15)		0.06^c^ (0.03 to 0.09)		0.06^c^ (0.03 to 0.09)	
	Health literacy, B (95% CI)	0.06^c^ (0.02 to 0.11)		0.06^c^ (0.01 to 0.10)		0.04^c^ (0.00 to 0.09)		0.02 (−0.02 to 0.06)	
	*R*^2^ (adjusted *R*^2^)	0.02 (0.01)^c^		0.04 (0.03)^c^		0.02 (0.02)^c^		0.02 (0.01)^c^	

^a^All effects were measured on a scale ranging from 1 to 7.

^b^B: unstandardized coefficient.

^c^Significant value (*P*<.05).

^d^ES: education status.

^e^GP: general practitioner.

^f^NL: the Netherlands.

**Table 5:** Multiple linear regression analyses of determinants for the effects of online access to medical records (better communication, informed decision-making [IDM] information, IDM values, and IDM making decisions; N=2402).

Variable			Better communication		IDM^a^ information		IDM values		IDM making decisions
Score^b^, mean (SD)			5.00 (1.21)		5.17 (1.17)		5.22 (1.16)		5.22 (1.17)
**Model 1**									
	Online access, B^c^ (95% CI)	0.24^d^ (0.14 to 0.34)		0.13^d^ (0.03 to 0.22)		0.16^d^ (0.06 to 0.26)		0.19^d^ (0.09 to 0.29)	
	*R*^2^ (adjusted *R*^2^)	0.01 (0.01)^d^		0.00 (0.00)^d^		0.00 (0.00)^d^		0.01 (0.01)^d^	
**Model 2**									
	Online access, B (95% CI)	0.22^d^ (0.12 to 0.33)		0.11^d^ (0.01 to 0.21)		0.14^d^ (0.04 to 0.24)		0.16^d^ (0.06 to 0.26)	
	Female vs other, B (95% CI)	0.21 (−0.75 to 1.17)		0.00 (−0.93 to 0.93)		−0.24 (−1.16 to 0.69)		0.28 (−0.65 to 1.22)	
	Female vs male, B (95% CI)	0.03 (−0.07 to 0.13)		−0.04 (−0.14 to 0.05)		−0.06 (−0.16 to 0.03)		−0.04 (−0.13 to 0.06)	
	Low ES^e^ vs medium ES, B (95% CI)	0.05 (−0.07 to 0.17)		0.09 (−0.02 to 0.21)		−0.03 (−0.15 to 0.08)		−0.01 (−0.12 to 0.11)	
	Low ES vs high ES, B (95% CI)	0.02 (−0.12 to 0.16)		0.04 (−0.10 to 0.18)		−0.07 (−0.21 to 0.06)		−0.05 (−0.18 to 0.09)	
	No vs minimum of one visit to the GP^f^ due to a mental health complaint, B (95% CI)	−0.02 (−0.12 to 0.08)		−0.05 (−0.15 to 0.05)		−0.04 (−0.13 to 0.06)		−0.05 (−0.15 to 0.05)	
	No vs minimum of one chronic disease, B (95% CI)	−0.07 (−0.18 to 0.03)		−0.02 (−0.12 to 0.08)		0.00 (−0.10 to 0.10)		0.02 (−0.08 to 0.12)	
	Born in NL^g^ vs migrant, B (95% CI)	0.10 (−0.13 to 0.34)		0.20 (−0.03 to 0.43)		0.13 (−0.09 to 0.36)		0.20 (−0.02 to 0.43)	
	Age, B (95% CI)	0.00^d^ (0.00 to 0.01)		0.00 (0.00 to 0.00)		0.00^d^ (0.00 to 0.01)		0.00^d^ (0.00 to 0.01)	
	Digital literacy, B (95% CI)	0.07^d^ (0.04 to 0.10)		0.07^d^ (0.04 to 0.10)		0.08^d^ (0.05 to 0.11)		0.08^d^ (0.05 to 0.11)	
	Health literacy, B (95% CI)	0.04 (0.00 to 0.08)		0.05^d^ (0.01 to 0.09)		0.03 (−0.01 to 0.07)		0.03 (−0.01 to 0.07)	
	*R*^2^ (adjusted *R*^2^)	0.02 (0.02)^d^		0.02 (0.01)^d^		0.02 (0.02)^d^		0.02 (0.02)^d^	

^a^IDM: informed decision-making.

^b^All effects were measured on a scale ranging from 1 to 7.

^c^B: unstandardized coefficient.

^d^Significant value (*P*<.05).

^e^ES: education status.

^f^GP: general practitioner.

^g^NL: the Netherlands.

#### Confounders

Across most effects, differences in ratings were associated with digital and health literacy. Participants with higher digital literacy as well as those with higher health literacy indicated more strongly that online access would cause *practical changes in their GP health care*, specifically a better overview of health care and appointments, an increased ability to correct mistakes in the medical record, and more time investment in health care. Additionally, the belief that online access leads to more personal contact with the GP or the practice staff, and more telephone calls with the GP or the practice assistant was stronger for participants with higher health literacy. Participants with higher digital literacy more strongly believed that online access to medical records would increase their ability to prepare consultations with the GP.

Participants with higher digital literacy as well as those with higher health literacy also rated most *improvements* *in affective outcomes and the GP-patient relationship* more strongly (ie, feeling less overwhelmed, feeling less anxious, more patient involvement, and more equal-feeling conversations with the GP). Further, participants with higher digital literacy more strongly perceived the effect of better communication with the GP from online access to medical records.

Lastly, the effect on *informed decision-making* was perceived more strongly by both participants with higher digital literacy and those with higher health literacy, specifically the effect of having more information to make decisions about their health. Perceptions that online access to medical records can increase the ability to make decisions about health that align with own values and can increase the ability to make decisions about health in general were higher for participants with higher digital literacy.

## Discussion

This study showed robust effects of the use of online access to medical records on patient empowerment, the GP-patient relationship, and informed decision-making, even when taking sociodemographic factors and patient characteristics into account. The results align with commonly envisioned effects of online access [1]. By supporting informed decision-making, online access to medical records might help to prevent decisional conflict for patients [11,12] and thereby foster treatment adherence [13]. Online access might have the potential to increase patient satisfaction, as patient empowerment and involvement have been linked to patient outcomes, such as patient satisfaction, previously [46]. However, effect sizes in our study were small. There are several possible explanations and implications.

First, the impact of online access to medical records on patient empowerment, the GP-patient relationship, and informed decision-making might not be that large. This might be in line with the observation of several reviews that research to date fails to provide strong evidence that online access to medical records improves the patient-provider relationship and empowers patients [17,21].

Second, a time period of 6 months between the measurement of online access use and effects might have been too short for the effects to unfold completely. The rates of provision and use of online access have only recently been rising noticeably since the introduction of the statuary right for patient access in 2020. According to the inclusion criteria, all participants in this study had contact with their GP at least once in the 6 months prior to the study. However, it might still be that participants did not make use of GP health care or online access to medical records enough to experience noticeable effects in their health care process yet. The small effects after 6 months could indicate a trend that might continue in the future. This implies that to monitor how effects develop, repeated measurements over a longer time period are warranted.

Third, there might be other factors moderating the effects. An umbrella review on the current state of evidence regarding patient portals suggests that differences in local organizational contexts, such as the attitude of health care providers, might be a reason for incongruent effect findings [17]. Similarly, provider encouragement for use was found to be crucial for long-term [47] and meaningful [48] use. Especially in this early phase of online access implementation, the strength of effects for patients might considerably depend on the attitudes of GPs and practice staff toward online access. Contrary to patients’ expectations of fewer telephone calls [14], GPs’ worries about [17] and experiences of [49] increases in workload are supported by our finding that compared with nonusers, users indicated more strongly that online access to medical records would lead to more personal contact, consultations, and telephone contact with the GP or practice assistant. For online access to improve health care system functioning and efficiency, provider perspectives, and especially potential concerns about increases in workload, have to be explored and addressed. We would like to inform policy makers that to unlock the potential of patient online access to medical records, the implementation should be accompanied, or better still preceded, by an investigation of the optimal conditions and corresponding strategies that facilitate a positive impact for patients.

Digital literacy and health literacy were associated with differences in ratings across almost all effects (eg, participants with higher digital or health literacy scores rated the potential impact of online access to medical records on having a better overview of their health care more strongly than participants with lower scores). Thus, the benefits of online access to medical records might be unevenly distributed. Generally, patients with lower health literacy rate their health as worse [50]. Our results suggest that while health care demand is the highest among this group, they are less likely to benefit from online access. As members of an internet research panel, participants in our study presumably had above-average digital skills, as reflected in the high score on digital literacy (Table 1). However, at the same time, this high score might be representative of the Dutch population, where almost 80% were classified as having basic or high digital skills in 2019 [51]. Introduction of new digital health care tools should not lead to a relative disadvantage for groups that are already more vulnerable. Possibly, additional training for vulnerable groups or improvements of accessibility at the portal level could address this issue [52], but additional research is needed on the needs of patient groups with low digital and health literacy [28]. As long as disparities in the benefits of online access to medical records (and other digital health care tools) persist, nondigital options should remain available for all actions in the health care management of patients.

While it is important to take advantage of the knowledge gained from researchers worldwide, international perspectives highlight the influence of social and cultural factors on patients’ use of online access to medical records and the potential effects of this access [53]. Those appear to differ across patient populations in different geographical areas, sociocultural contexts, and stages of online access implementation [53]. Consequently, we concluded that the generalizability of our results and the applicability of implications are particularly relevant and likely limited to countries that have sociocultural contexts and technical infrastructure similar to the Netherlands.

A strength of the study is that in addition to building on insights from previous research conducted in different contexts and countries, the inclusion of variables measuring effects was informed by preceding local qualitative research. Thereby, we were able to include insights into the possible effects of online access to medical records from the Dutch perspective. Another strength is that the large sample size enabled detection of even small effects, which might have remained undetected in a smaller sample. Thereby, we might have discovered trends and laid the foundation for future effect monitoring.

It should be noted that we measured effects by comparing users’ and nonusers’ beliefs about the impact of online access to medical records on specific outcome measures. Thus, for both groups, participants had to attribute possible changes in outcome measures themselves to the use of online access. For nonusers, this might have been more challenging, but because our goal was specifically to examine patient perceptions, we believe the measurement is appropriate for our research.

As the gender (identity) group “nonbinary/another gender” had only 6 participants, we cannot draw conclusions from the regression coefficients for this gender category. The wide confidence intervals reflect this inaccuracy of estimates. However, precisely because of the very small proportion of this group in the total sample, this most likely has no influence on the rest of the results.

The findings of this study can inform impact evaluations as well as strategies that provide patients with information about possible effects that they can consider in their decisions about whether to use online access to their medical records.

Online access to medical records has the potential to empower patients, increase patient participation in health care, and foster informed decision-making. The effects in this study were small but might grow over time. Thus, monitoring the development of effects is advised. Differences in ratings across almost all effects that were associated with digital and health literacy indicated that the potential benefits of online access might be unevenly distributed. Future research should explore the needs of vulnerable patient groups, the conditions under which online access to medical records might have positive effects for patients, and the impact of online access to medical records on providers’ workload. This knowledge will help to prevent disparity in effect distribution and possibly facilitate the improvement of health care system functioning and efficiency. Additionally, the results from this study can inform impact evaluation and support individual patients in their decisions about whether to use online access to their medical records.

## Appendix

Multimedia Appendix 1 Survey items assessing beliefs about online access to medical records.

## Abbreviations

GP: general practitioner
OSF: Open Science Framework

## Data Availability

The data in this study are classified as pseudonymized and thus subject to the European Union General Data Protection Regulation (GDPR). Therefore, the data sets generated and analyzed during this study are not publicly available but are available from the corresponding author on reasonable request. Under contractually ensured guarantee of adherence to the GDPR, the data can be shared.
