# Supplementary material for: The Effects of Online Access to General Practice Medical Records Perceived by Patients: Longitudinal Survey Study
Source: J Med Internet Res. 2023 Jun 2;25:e47659. doi: 10.2196/47659 (PMC10276325; doi:10.2196/47659)
Supplement: Multimedia Appendix 1 [file jmir_v25i1e47659_app1.docx]

## **Multimedia Appendix 1. Survey items assessing beliefs about online access to medical records.**

The following questions are about possible consequences of accessing your GP medical records online.

Do you not use online access? If so, when answering, think about the consequences you would expect if you used it.

Do you use online access? If so, when answering, think about experiences you have had.

By having online access...

1. … I have [much less/ much more] overview of my health care.

2. ... I have [much less/ much more] overview of my appointments.

3. ... I am [much less/ much more] able to ensure that mistakes in my medical record are corrected.

4. ... I feel [much less/ much more] overwhelmed.

5. ... I feel [much less/ much more] anxious.

6. ... I have [much less/ much more] personal contact with the GP and practice staff.

7. … I have [much fewer/ much more] consultations with the GP or practice assistant.

8. ... I have [much less/ much more] telephone contact with the GP or practice assistant.

9. ... I invest [much less/ much more] time in my health care.

10. ... I feel [much less/ much more] involved in my own health care.

11. … conversations with my GP feel [much more unequal/much more equal].

12. … I can prepare my consultations with the GP [much worse/much better].

13. … communication with my GP becomes [much worse/much better].

14. ... I have [much less/much more] information for making decisions about my health.

15. ... I am [much less/much more] able to make decisions about my health that suit me and what I think is important.

16. ... I am [much less/much more] able to make decisions about my health.

[All answer scales range from 1 to 7]
